# Decreased Pyruvate but Not Fatty Acid Driven Mitochondrial Respiration in Skeletal Muscle of Growth Restricted Fetal Sheep

**DOI:** 10.3390/ijms242115760

**Published:** 2023-10-30

**Authors:** Weicheng Zhao, Amy C. Kelly, Rosa I. Luna-Ramirez, Christopher A. Bidwell, Miranda J. Anderson, Sean W. Limesand

**Affiliations:** 1 School of Animal and Comparative Biomedical Sciences, University of Arizona, Tucson, AZ 85719, USA; weichengz@arizona.edu (W.Z.); amyk1@arizona.edu (A.C.K.); rosaluna@arizona.edu (R.I.L.-R.); miranda1@arizona.edu (M.J.A.); 2 Department of Animal Sciences, Purdue University, West Lafayette, IN 47907, USA; cbidwell@purdue.edu

**Keywords:** intrauterine growth restriction, mitochondria, skeletal muscle, fetus, developmental programming

## Abstract

Fetuses with intrauterine growth restriction (FGR) have impaired oxidative and energy metabolism, with persistent consequences on their postnatal development. In this study, we test the hypothesis that FGR skeletal muscle has lower mitochondrial respiration rate and alters the transcriptomic profiles associated with energy metabolism in an ovine model. At late gestation, mitochondrial oxygen consumption rates (OCRs) and transcriptome profiles were evaluated in the skeletal muscle collected from FGR and control fetuses. The ex vivo mitochondrial OCRs were reduced *(p* < 0.01) in permeabilized FGR soleus muscle compared to the control muscle but only with pyruvate as the metabolic substrate. Mitochondrial OCRs were similar between the FGR and control groups for palmitoyl-carnitine (fatty acid-driven) or pyruvate plus palmitoyl-carnitine metabolic substrates. A total of 2284 genes were differentially expressed in the semitendinosus muscle from growth restricted fetuses (false discovery rate (FDR) ≤ 0.05). A pathway analysis showed that the upregulated genes (FGR compared to control) were overrepresented for autophagy, HIF-1, AMPK, and FOXO signaling pathways (all with an FDR < 0.05). In addition, the expression of genes modulating pyruvate’s entry into the TCA cycle was downregulated, whereas the genes encoding key fatty acid oxidation enzymes were upregulated in the FGR muscle. These findings show that FGR skeletal muscle had attenuated mitochondrial pyruvate oxidation, possibly associated with the inability of pyruvate to enter into the TCA cycle, and that fatty acid oxidation might compensate for the attenuated energy metabolism. The current study provided phenotypic and molecular evidence for adaptive deficiencies in FGR skeletal muscle.

## 1. Introduction

Neonates born with fetal growth restriction (FGR) have lower birth weights and increased risks for developing metabolic diseases in adulthood, such as type 2 diabetes, obesity, and cardiovascular diseases [1,2,3]. Fetal growth restriction is commonly associated with placental insufficiency which deprives the developing fetus of oxygen and nutrients [4,5]. One of the fetal consequences of placental insufficiency is the reduction in blood perfusion to peripheral tissues, including the skeletal muscle, in order to sustain oxygen and nutrient supplies to other key organs, such as the heart and brain [6,7]. Therefore, skeletal muscle is likely to be the primary target for tissue-specific growth restrictions under placental insufficiency conditions [8]. Evidence from animal studies shows that FGR fetuses exposed to placental insufficiency have lower body and muscle weights, impaired muscle growth rates, and asymmetrical fetal growth [7,9,10,11]. In addition, these deficits in skeletal muscle growth and metabolic dysfunction persist during postnatal growth with FGR progeny having a limited capacity for postnatal muscle growth [12]. For example, FGR offspring had a lower protein content, reduced lean tissue deposition, and increased carcass adiposity in pigs and lambs [13,14,15,16].

In response to placental insufficiency, FGR fetuses slow protein accretion rates for new tissues to preserve energy for basal metabolic requirements [17]. Despite this adaptation, whole-body fractional glucose oxidation rates were reduced in FGR fetuses [18,19]. The impaired energy metabolism of FGR fetuses might be associated with defects in the mitochondrial tricarboxylic acid (TCA) cycle and subsequently oxygen-dependent oxidative phosphorylation, which is the primary cellular ATP synthesis pathway. Under hypoxic conditions, cells will switch their primary metabolic strategy away from oxidative phosphorylation to oxygen-independent glycolysis so that the minimum level of bioenergetic homeostasis is maintained [20]. Studies showed that TCA cycle enzyme abundance and mitochondrial electron transport chain (ETC) activity were lower in skeletal muscle from FGR fetal sheep [11,21]. Consequently, impaired muscle oxidative metabolism might explain the reduced oxygen uptake, impaired myogenesis, and compromised muscle hypertrophy that manifest in FGR muscle [7,10,22].

Although there is evidence that growth restricted fetuses have impaired global oxidative metabolism, the metabolic adaptation in skeletal muscle mitochondria function and the underlying molecular mechanisms are less understood. Therefore, we proposed to test the hypothesis that FGR fetal sheep had impaired skeletal muscle ex vivo mitochondrial oxygen consumption rates. In addition, a comparative transcriptomic analysis (RNA-seq) was conducted to identify the molecular mechanisms governing the metabolic adaptation in FGR skeletal muscle.

## 2. Results

### 2.1. Fetal and Placental Morphometry

At late gestation (gestational age of 133 ± 1 days), fetal weights were lower in the FGR group (*n* = 9) compared to those of the control (CON, *n* = 12) (2087 ± 204.9 vs. 3138 ± 141.5 g, *p* < 0.01). Placentae from the FGR group weighted 59% less than those from the CON group (137 ± 9.5 vs. 336 ± 18.1 g, *p* < 0.01). The FGR fetuses had lower semitendinosus (3.9 ± 0.38 vs. 6.3 ± 0.38 g, *p* < 0.01) and biceps femoris (11.1 ± 1.36 vs. 17.2 ± 0.87 g, *p* < 0.01) muscle weights. In addition, the FGR fetuses tended to have lower semitendinosus muscle weights normalized to fetal weights (0.188 ± 0.006 vs. 0.201 ± 0.007%, *p* = 0.09). However, the relative biceps femoris weights normalized to fetal weights were not different between the CON and FGR groups (0.53 ± 0.016 vs. 0.55 ± 0.012%, *p* > 0.1).

### 2.2. Mitochondria Oxygen Consumption Rates

**Figure 1:**
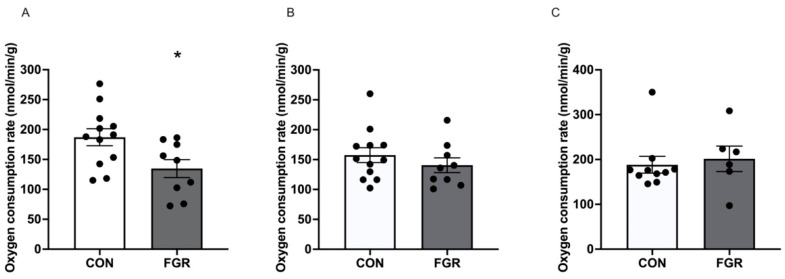
Permeabilized soleus muscle mitochondrial oxygen consumption rates between control (CON; *n* = 10 to 12) and fetal-growth-restricted (FGR; *n* = 6 to 9) groups with (**A**): glucose substrate (5 mM pyruvate + 1 mM malate + 2 mM ADP); (**B**): palmitoyl-carnitine substrate (0.2 mM palmitoyl-carnitine + 1 mM malate + 2 mM ADP); and (**C**): pyruvate plus palmitoyl-carnitine substrate (5 mM pyruvate + 1 mM malate + 2 mM ADP + 0.2 mM palmitoyl-carnitine). * *p* < 0.05.

Permeabilized soleus muscle mitochondrial OCRs with pyruvate (a glucose-derived metabolic substrate) were reduced (*p* = 0.027) in the FGR compared to the CON group (Figure 1). However, muscle mitochondrial OCRs were similar between the FGR and CON groups for the palmitoyl-carnitine (a fatty acid-derived metabolic substrate) or pyruvate plus palmitoyl-carnitine substrate groups (Figure 1).

### 2.3. Differentially Expressed Genes, Gene Ontology and Pathway Enrichment Analyses

**Figure 2:**
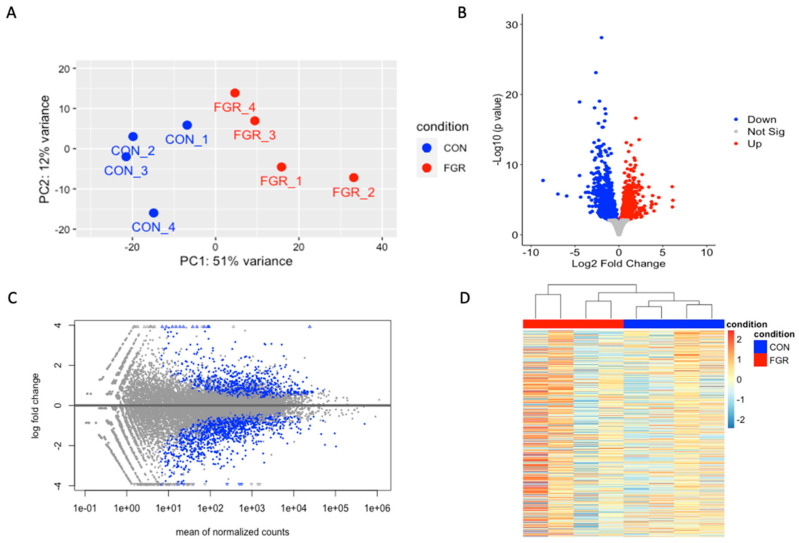
Transcriptome analysis of FGR skeletal muscle. Next-generation RNA sequencing results for semitendinosus muscle from control fetuses (CON, *n* = 4) and growth restricted fetuses (FGR, *n* = 4) were compared. (**A**): PCA plots were obtained based on count matrix, and the plot showing variance of the biological replicates in CON and FGR groups. (**B**): Volcano plot of genes detected via RNA-seq analysis; the differentially expressed genes (FGR compared to CON) are denoted with red (upregulated), blue (downregulated), and grey (not different) dots. (**C**): MA plot presents the mean of normalized counts against log2 fold changes. Genes with an adjusted *p* value less than 0.05 are shown in blue. (**D**): Heatmap of the top 500 differentially expressed genes is presented.

A total of 2284 genes were differentially expressed (FDR < 0.05) between the FGR and CON fetal skeletal muscle (Figure 2). Among the 2284 differentially expressed genes (DEGs), 1032 genes were upregulated, and 1252 genes were downregulated in the FGR compared to the control fetuses. Table 1 lists a subset of DEGs containing genes with the greatest fold change (up- and downregulated) and the metabolic genes of interest that we particularly focused on in our study.

**Figure 3:**
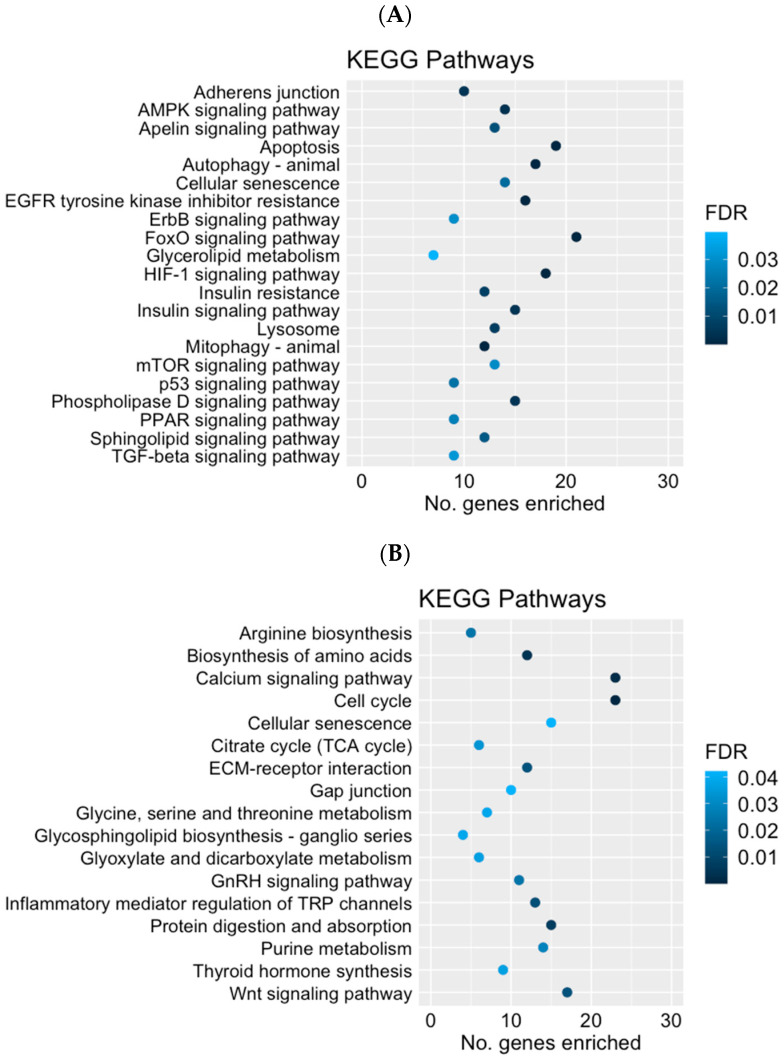
Kyoto Encyclopedia of Genes and Genomes (KEGG) pathway terms significantly enriched (FDR ≤ 0.05) by the upregulated (**A**) and downregulated (**B**) differentially expressed genes (FGR compared to CON).

An gene ontology overrepresentation analysis was performed for the up- and downregulated genes separately. For the upregulated genes (FGR compared to CON), the HIF-1 signaling pathway (oas04066), the AMPK signaling pathway (oas04152), autophagy (oas04140), mitophagy (oas04137), the FoxO signaling pathway (oas04068), and insulin resistance (oas04931) were among the KEGG pathways that were enriched (all FDRs < 0.01) (Figure 3A). In addition to the KEGG pathway, gene ontology (GO) terms for the positive regulation of angiogenesis (GO:0045766) were enriched (FDR = 0.002) with the upregulated DEG. Among the downregulated genes (FGR compared to CON), the cell cycle (oas04110, FDR < 0.001), citrate cycle (TCA cycle, oas00020, FDR = 0.04), calcium signaling pathway (oas04512, FDR = 0.002), and biosynthesis of amino acid (oas01230, FDR = 0.004) were among the enriched KEGG pathways (Figure 3B). There were no GO terms significantly enriched by the downregulated genes. The full list of the enriched pathways and the GO terms with their associated genes, gene ontology ID, and FDRs are presented in Supplementary Table S1.

### 2.4. Validation of the RNA-Seq Data via qPCR Experiments

**Figure 4:**
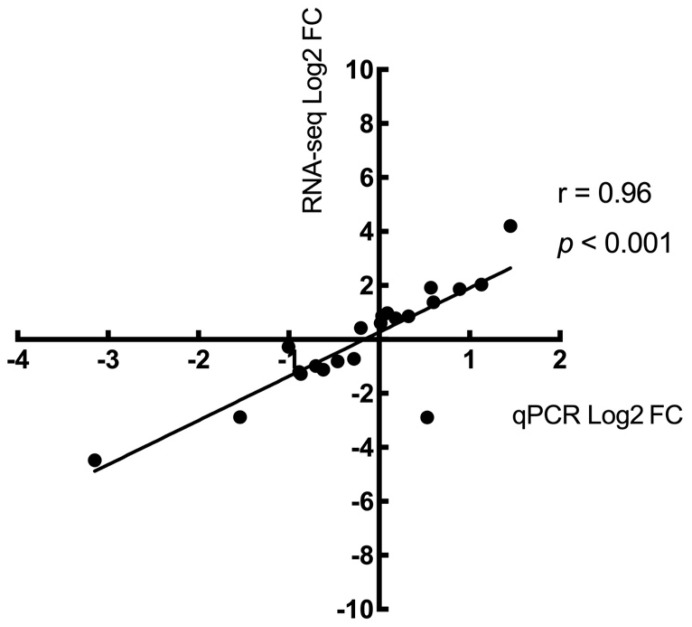
Correlation of transcript expression levels in RNA-seq and qPCR experiments. Expression levels (log2 fold change) were calculated for 20 candidate genes. Results from the RNA-seq experiment are positively correlated (*p* < 0.001) with log2 fold results determined by qPCR methods. The Pearson correlation coefficient (r) was 0.96, with a 95% confidence interval of 0.907 to 0.986.

Twenty candidate DEGs that were among the top fold changes and were associated with the FoxO signaling pathway, the AMPK signaling pathway, and fatty acid oxidation were chosen for our qPCR experimental validation. The log2 fold changes identified between the RNA-seq and qPCR experiments are plotted in Figure 4. There was a positive correlation (r = 0.96, *p* < 0.001) for the log2 fold changes in candidate genes between these two experiments, showing the validation of the RNA-seq findings.

## 3. Discussion

**Figure 5:**
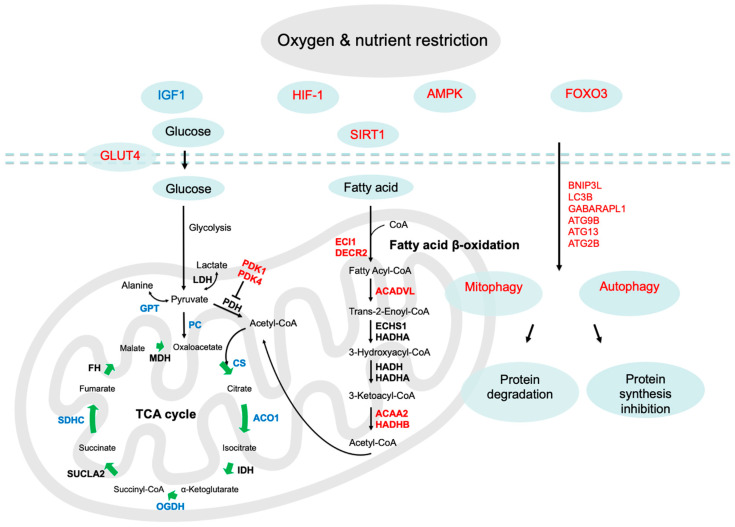
Summary of the differentially expressed genes and signaling pathways identified in the current study. The up- and downregulated (FGR compared to CON) pathways and genes are indicated in red and blue, respectively, whereas genes and pathways that are not different from controls are in black. Functional gene enrichment of upregulated genes identified significant perturbations in AMPK, HIF-1, and FOXO signaling pathways, indicating the FGR skeletal muscle was under hypoxic and cellular stress. Activation of stress-sensing pathways promotes catabolism as evidenced by greater expression of genes involved in autophagy-lysosomal proteolysis. Pyruvate’s entry into the TCA cycle is achieved via its conversion to either acetyl-CoA or oxaloacetate, catalyzed by pyruvate dehydrogenase (PDH) or pyruvate carboxylase (PC), respectively. Lower abundance of PC transcripts and higher abundance of PDK1 and PDK4 transcripts indicate that pyruvate’s entry into the TCA cycle was reduced. These findings parallel the lower pyruvate-driven oxygen consumption rates (OCRs) in skeletal muscle mitochondria. Higher expression of fatty acid oxidation enzyme genes and a normal fatty acid-driven mitochondrial OCR imply that fatty acid oxidation may compensate for the attenuated pyruvate-driven mitochondrial respiration. IGF1, insulin-like growth factor 1; AMPK, 5′ AMP-activated protein kinase; FOXO3, forkhead box O3, GLUT4, glucose transporter 4; SIRT1, NAD-dependent deacetylase sirtuin-1; BNIP3L, BCL2 interacting protein 3 like; GABARAPL1, GABA type A receptor associated protein like 1; LC3B, microtubule-associated proteins 1A/1B light chain 3B; ATG: autophagy related; PC, pyruvate carboxylase; GPT, glutamic-pyruvic transaminase (alanine aminotransaminase); LDH, lactate dehydrogenase; PDK, pyruvate dehydrogenase kinase; PDH, pyruvate dehydrogenase; CS, citrate synthase; ACO1, aconitase 1; DIH, isocitrate dehydrogenase; OGDH, oxoglutarate dehydrogenase; SUCLA2, succinate-CoA ligase ADP-forming subunit B; SDHC, succinate dehydrogenase subunit C; FH, fumarate hydratase; MDH, malate dehydrogenase; ECI1: enoyl-CoA delta isomerase 1; DECR2: 2,4-dienoyl-CoA reductase 2; ACADVL, very-long-chain acyl-CoA dehydrogenase; ECHS1, short-chain enoyl-CoA hydratase; HADHA, mitochondrial trifunctional protein, alpha subunit; HADHB, mitochondrial trifunctional protein, beta subunit; HADH, short-chain (S)-3-hydroxyacyl-CoA dehydrogenase; ACAA2, Medium-chain 3-ketoacyl-CoA thiolase.

In the present study, we used an ovine model of FGR to demonstrate that FGR fetuses had lower ex vivo mitochondrial oxygen consumption rates in permeabilized soleus muscle when glucose-derived pyruvate was the metabolic substrate. FGR muscle had a normal mitochondrial OCR when exposed to fatty acids (palmitoyl-carnitine) or pyruvate plus fatty acid substrate pairs. Our further exploration of the skeletal muscle molecular adaptation showed that the lower pyruvate oxidation was partially explained by lower pyruvate carboxylase (PC) and citrate synthase (CS) mRNA expression, and higher pyruvate dehydrogenase kinase (PDK) mRNA expression which may prevent pyruvate’s entry into the TCA cycle for subsequent oxidative respiration. We also showed lower expression of genes encoding TCA cycle enzymes (PC, CS, ACO1, SDHC, and OGDH) in FGR muscle (Figure 5), which is supported by our previous results of reduced protein abundance (PC, SDHC, and OGDH) in FGR muscle mitochondria [11]. Our findings also indicate that FGR skeletal muscle was in a catabolic state, which was evidenced by the upregulation of genes (e.g., FOXO3, BNIP3L, LC3B, and GABARAPL1) and pathways (autophagy and mitophagy) related to proteolysis (Figure 5).

Permeabilized soleus muscle fibers from FGR fetuses have lower mitochondrial OCRs with pyruvate as the substrate but were not different from those with fatty acids as the substrate. These data indicate that deficiencies reside in pyruvate’s entry into the mitochondria rather than TCA cycle deficiencies because acetyl-CoA derived from fatty acids had a normal OCR in FGR muscle. Upon its transport into the mitochondria, pyruvate enters into the TCA cycle via its conversion to either acetyl-CoA or oxaloacetate, catalyzed by pyruvate dehydrogenase (PDH) or PC, respectively. Both acetyl-CoA and oxaloacetate are further catalyzed by CS, the first enzyme in the TCA cycle. Supporting the current data on lower pyruvate oxidative respiration, we observed lower expression of genes encoding PC and CS. In addition, there was higher expression of genes encoding PDK1 and PDK4, which phosphorylate and inactivate PDH. The current data are also supported by our previous proteomic data showing the downregulation of mitochondrial pyruvate carrier 2 (MPC2) and PC which governs pyruvate’s flux into mitochondria and the TCA cycle [11]. Another possible explanation for the inability of pyruvate’s entry into the TCA cycle in FGR muscle is that pyruvate might be used for alanine or lactate production as an adaptation to meet energy needs [23]. We observed lower expression of the genes (GPT and GPT2) encoding glutamic-pyruvate transaminase, also known as alanine aminotransaminase (ALT), which catalyzes the reversible conversion between alanine and pyruvate. Downregulated alanine aminotransaminase gene expression suggests the inhibition of the conversion from alanine to pyruvate, leading to increased alanine release from muscle and intramuscular pyruvate accumulation [23]. However, the current data did not identify gene expression changes in lactate dehydrogenase (LDH), which catalyzes pyruvate to lactate, despite increased plasma lactate concentrations in FGR fetuses observed in our previous studies [10,24]. The current transcriptomic data also showed that the uncoupling protein genes UCP1 and UCP2 were upregulated in FGR muscle, suggesting mitochondrial uncoupled respiration and a lower ATP content [25]. Together, these results indicate that FGR muscle had lower pyruvate-driven mitochondrial respiration, which was associated with the downregulation of enzymes involved in pyruvate’s entry into the TCA cycle.

In FGR muscle, a lower OCR with pyruvate but not with pyruvate plus fatty acid substrates indicates that fatty acid oxidation was increased when pyruvate oxidation was attenuated under the condition that both substrates are supplemented ex vivo. Although fetal fatty acid oxidation rates are low and are not deemed to be a major energy source for the fetus under normal circumstances [26], the expression levels of the fatty acid oxidation enzyme genes were greater in the skeletal muscle of neonates born with fetal growth restriction [27]. Herein, several genes encoding key fatty acid oxidation enzymes were higher, including the acyl-CoA dehydrogenase, very-long-chain (ACADVL), mitochondrial trifunctional protein, betta unit (HADHB), and acetyl-CoA acyltransferase 2 (ACAA2) genes. These gene expression and OCR data indicate that the fatty acid oxidation compensated for the attenuated pyruvate oxidation. Similar to these findings, lower mitochondrial OCRs were observed in FGR skeletal muscle in sheep and rat models [11,28]. However, a recent study reported that FGR sheep had similar mitochondrial OCRs in permeabilized muscle fiber compared to control fetuses despite the lower protein expression of the mitochondrial complex subunits [21]. The reasons for this discrepancy in the muscle mitochondrial respiration across the studies remains unknown and requires further investigations.

An interesting finding is that the pathways associated with autophagy and mitophagy were upregulated in FGR muscle, suggesting increased proteolysis. Skeletal muscle proteolysis is largely driven by the autophagy-lysosomal pathway and the ubiquitin proteasome pathway [29]. We observed greater expression of autophagy-related genes (BNIP3L, GABARAPL1, and LC3B) in FGR muscle, which were associated with the formation of mitochondrial autophagosomes and protein turnover. For example, BNIP3L is localized at the outer membrane of the mitochondria, and the overexpression of BNIP3L induces mitochondrial remodeling and damage by binding to LC3B [30]. The excessive removal of dysfunctional mitochondria via mitophagy causes muscle loss. The upregulation of the proteolysis in FGR muscle might be associated with the amino acid release from skeletal muscle to support energy production in other tissues during the catabolic state, such as hepatic gluconeogenesis [23,31]. Similarly, the upregulation of protein breakdown gene markers and higher amino acid oxidation have been reported in chronically hypoglycemic fetuses [32,33], while other studies have shown that fetal total or hindlimb muscle protein breakdown rates were unaffected in FGR fetuses [7,34]. The mechanism augmenting the transcriptional targets for autophagy and proteolysis in FGR muscle is not fully understood, but it might be associated with cellular hypoxic stress caused by placental insufficiency-induced fetal hypoxemia. Numerous reports have shown that FGR fetuses have a lower arterial oxygen content [18,35]. Strikingly, FGR fetuses also have lower hindlimb oxygen and amino acid uptakes reflecting 30% less weight-specific oxygen consumption in fetal muscle [7]. Low cellular oxygen and energy utilization promote the hypoxia signaling and AMPK signaling pathways [29,36], as evidenced in our results (Figure 3A). The upregulation of oxygen and energy-sensing pathways activated FOXO3 [29], which is known to induce autophagy and protein breakdown [37]. In the absence of an adequate oxygen supply, there is the HIF-dependent suppression of mitochondrial oxidative phosphorylation whereas cell anaerobic glycolysis is upregulated to maintain bioenergetic requirements [38]. We observed increased expression of GLUT4, a major muscle glucose transporter, in FGR muscle, indicating that there was a maintained or increased peripheral glucose uptake and utilization in the presence of a low-fetal-glucose and -oxygen environment [18]. However, the inability of pyruvate to flux into the mitochondria and the TCA cycle, evidenced by the lower pyruvate-driven oxidative respiration and the downregulation of related transcription, reinforced the fact that the FGR fetuses had limited oxygen-dependent glucose oxidation to ensure their survival [18,19].

A limitation of this study is that evidence from the transcriptomic level was explored while changes in protein abundance were beyond its scope. Future studies are warranted to validate whether these gene expression changes can be translated to functional and protein changes, although the parts of gene markers identified in the current study have been validated at the protein and functional level in recent studies from our lab and others [11,21].

## 4. Materials and Methods

### 4.1. Ethical Approval

All experiments on animals were approved by the Institutional Animal Care and Use Committee at The University of Arizona (Protocol #08-132), which is accredited by the American Association for Accreditation of Laboratory Animal Care International (AAALAC).

### 4.2. Animals and Experimental Design

Fetal muscle samples were obtained from a subset cohort of animals used in previously published studies [24,35]. Pregnant ewes (Columbia/Rambouillet crossed; 54 ± 2 kg) with singleton pregnancies were obtained from the University of Arizona Sheep Unit (Tucson, AZ, USA) and transported to the laboratory at 35 ± 2 days of gestation age (dGA; term 149 dGA). After confirmation of pregnancy with ultrasonography, ewes were assigned to one of two experimental groups: control and FGR. Fetuses with growth restriction were produced by exposing pregnant ewes to elevated ambient temperatures (40 °C for 12 h; 35 °C for 12 h; dew point 22 °C) from 40 ± 1 to 90 ± 1 dGA. Control fetuses were taken from pregnant ewes maintained at constant 25 °C that were pair fed to the average feed intake of ewes in the FGR group. All sheep were given ad libitum access to water and salt. Ewes and fetuses were euthanized at 133 ± 1 dGA (0.9 pregnancy) for sample collection. Animals were euthanized with an overdose of sodium pentobarbital (86 mg kg^−1^) and phenytoin sodium (11 mg kg^−1^; Euthasol; Virbac Animal Health, Fort Worth, TX, USA). Fetal and placental gross morphometry was conducted postmortem following dissection. Placental and fetal weights were measured and recorded. Fetal semitendinosus muscle was removed, weighed, and immediately frozen in liquid nitrogen. The soleus was dissected from the hindlimb and transferred into cold phosphate buffered saline for fiber dissection and further analysis.

### 4.3. Permeabilized Muscle Mitochondrial Oxygen Consumption Rate

Oxygen consumption rates were measured in the permeabilized soleus muscle as per the protocol previously described with modifications [39]. Briefly, fresh muscle fiber strips were separated from the soleus muscle biopsies (CON, *n* = 12 (7 females and 5 males); FGR, *n* = 9 (6 females and 3 males)). The muscle fibers were then permeabilized with saponin (50 μg/mL) in the BIOPS solution (containing 10 mM Ca-EGTA, 0.1 μL free calcium, 20 mM imidazole, 20 mM taurine, 50 mM K-MES, 0.5 mM DTT, 6.56 mM MgCl_2_, 5.77 mM ATP, and 15 mM phosphocreatine). The permeabilized muscle fibers were thoroughly rinsed in the respiration buffer containing 0.5 mM EGTA, 20 mM HEPES, 3 mM MgCl_2_·6H_2_O, 10 mM KH_2_PO_4_, 20 mM taurine, and 110 mM mannitol. Mitochondrial respiration for the permeabilized soleus muscle fibers was measured in a Fluorescence Lifetime Micro Oxygen Monitoring System (Instech Laboratories, Inc., Plymouth Meeting, Plymouth, PA, USA) with the following substrates added: the pyruvate substrate (5 mM pyruvate + 1 mM malate + 2 mM ADP), the fatty acid substrate (0.2 mM palmitoyl-carnitine + 1 mM malate + 2 mM ADP), or the pyruvate + fatty acid substrate (5 mM pyruvate + 1 mM malate + 2 mM ADP + 0.2 mM palmitoyl-carnitine). Mitochondrial state 3 OCRs were determined from the slope of pO_2_ disappearance over time in a linear range and calculated using the solubility of oxygen at 37 °C [40]. Values for each animal are representative of triplicate measurements normalized to wet muscle weight (g) and presented as nM O_2_ consumed per minute per gram of tissue. Cytochrome C and cyanide controls were used to verify that the procedure of permeabilization did not impact mitochondrial function and are commonly used procedures in metabolic studies.

### 4.4. RNA Extraction and Sequencing

RNA (CON *n* = 4 (1 females and 3 males)), FGR *n* = 4 (1 females and 3 males)) was extracted from the semitendinosus muscle using the RNeasy Mini Kit (Qiagen, Santa Clarita, CA, USA) as per the manufacturer’s instructions. RNA concentration was determined by measuring absorbance at 260 and 280 nm with the NanoDrop ND-1000 Spectrophotometer (NanoDrop, Willmington, DE, USA). RNA integrity was evaluated with an Experion Automated Electrophoresis System (Bio-Rad Laboratories, Hercules, CA, USA), and samples that had RNA Quality Index values of 9.6 to 10 were used for downstream analyses. The RNA samples were then submitted to University of Arizona Genomics Core for RNA-sequencing (RNA-seq). Messenger RNA (mRNA) was selected and double-stranded cDNA libraries with ligated sequencing adapters were constructed with the Illumina TruSeq RNA Sample Prep Kit (Illumina Inc., San Diego, CA, USA). Cluster generation was conducted with Illumina TruSeq 100bp PE (paired end) cluster kit before running it on the Illumina HiSeq2500 platform (Illumina Inc., San Diego, CA, USA).

### 4.5. Sequencing Data Reprocessing and Analysis

Quality control of sequencing reads was performed using FastQC (Babraham Bioinformatics, Cambridge, UK). The paired-end read numbers ranged from 33 to 48 million and were aligned to the sheep reference genome *Oar_rambouillet_v1.0* using STAR version 2.7.3a [41]. The average alignment rate across the samples was 82.8% ± 0.05 (SD). The aligned reads per gene were then counted using FeatureCount version 2.0.0 [42]. Differentially expressed genes (DEGs) were determined using DESeq2 package version 1.40.2 [43] in R version 4.3.1 [44]. Log fold change shrinkage function included in the DESeq2 package was performed to improve variability in genes with low expression. Genes with an adjusted *p* value or false discovery rate (FDR) ≤ 0.05 were considered as differentially expressed. Candidate differentially expressed genes were then subjected to gene ontology and pathway enrichment analyses (statistical overrepresentation tests) in KOBAS dataset version 3.0 [45], annotated for sheep. Upregulated and downregulated DEGs (FGR compared with CON) were analyzed separately. Gene ontology and pathway terms with an adjusted *p* value (FDR) ≤ 0.05 were considered enriched or overrepresented.

### 4.6. Quantitative PCR Validataion

Validation of RNA-seq data was performed via quantitative PCR (qPCR) on a larger cohort of CON (*n* = 11) and FGR (*n* = 11) ST muscle samples. Twenty candidate DEGs that were among the top fold changes and were associated with FOXO signaling, AMPK signaling, and fatty acid oxidation pathways were chosen for qPCR experimental validation. The qPCR assays were conducted in accordance with the guideline [46]. The oligonucleotide primer details are listed in Supplementary Table S2. Muscle RNA concentrations and integrity were tested as described above. RNA (1 µg/reaction) was reverse transcribed, in triplicate, into cDNA with Superscript III reverse transcription (Invitrogen, Carlsbad, CA, USA). Primer efficiencies were measured with serial cDNA dilutions. All primers had efficiency above 85%, and the threshold cycles (CTs) were linear over six orders of magnitude. Gene expression was determined via qPCR using SYBR Green (Qiagen) in an iQ5 Real-Time PCR Detection System (Bio-Rad Laboratories, Hercules, CA, USA). Samples were initially denatured (95 °C for 15 min) and then amplified with 45 cycles of denaturing (96 °C for 30 s), annealing (60–62 °C for 30 s), and fluorescence measurements during extension (72 °C for 10 s). Melt curves were performed after amplification to confirm product homogeneity. mRNA concentrations for each gene of interest were determined from triplicate cDNA reactions and normalized to the geometric mean of ribosomal protein RPS15, TBP, YWHAZ, and GAPDH.

### 4.7. Statistical Analysis

Skeletal muscle from male and female fetuses were included in all groups but effects of sex were not evaluated due to low numbers and because fetal sex was not a factor in previous studies [10,11]. Correlation in fold change of gene expression between RNA-seq and qPCR was determined with the Pearson correlation test in R. qPCR results were calculated as fold changes with the 2^−ΔΔCT^ method [47]. Mitochondrial oxygen consumption rate and qPCR data between two groups were compared using Student’s *t* test. Means are presented ± SEM. A *p* value < 0.05 was considered significant, and 0.5 ≤ *p* < 0.1 was considered a trend.

## 5. Conclusions

The findings from the current study demonstrated that the skeletal muscle from FGR fetuses near term had lower ex vivo mitochondrial oxygen consumption rates with pyruvate substrates. The lower respiration rates were potentially caused by the transcriptional regulation of enzymes that inhibited pyruvate’s entry into the TCA cycle (Figure 5). However, ex vivo respiration was not affected with fatty acid or pyruvate plus fatty acid substrates, suggesting that fatty acid oxidation might compensate for the attenuated pyruvate metabolism in FGR muscle. We also observed that the FGR muscle had upregulated oxygen and energy-sensing pathways that modulated the tissue towards catabolism as evidenced by increased gene markers of autophagy and mitophagy (Figure 5). However, the underlying mechanism for the upregulation of the proteolysis signaling is unclear and warrants further investigations. Overall, the present study provides phenotypic and molecular evidence for the adaptive defects in the skeletal muscle of FGR fetuses exposed to placental insufficiency.

## Figures and Tables

**Table 1:** A subset of differentially expressed genes (FGR compared to CON) containing genes with the highest fold changes and metabolic genes of interest that we particularly focused on in our study.

Gene Symbol	Full Gene Name	Log2 Fold Change
UCP1	Uncoupling protein 1	6.14
DPEP1	Dipeptidase 1	6.10
PIWIL3	Piwi-like RNA-mediated gene silencing 3	6.07
PDK4	Pyruvate dehydrogenase kinase 4	4.20
ARMC12	Armadillo repeat containing 12	3.78
NDUFA4L2	NDUFA4, mitochondrial complex associated like 2	2.21
ECI1	Enoyl-CoA delta isomerase 1	1.29
GABARAPL1	GABA type A receptor associated protein like 1	1.19
PDK1	Pyruvate dehydrogenase kinase 1	1.13
UCP2	Uncoupling protein 2	1.12
FOXO3	Forkhead box protein O3	0.97
MAP1LC3B	Microtubule associated protein 1 light chain 3 beta (LC3B)	0.91
EIF4EBP1	Eukaryotic translation initiation factor 4E binding protein 1	0.87
BNIP3L	BCL2 interacting protein 3 like	0.83
DECR2	2,4-dienoyl-CoA reductase 2	0.73
SLC38A3	Solute carrier family 38 member 1 (SNAT1)	0.69
NEDD4L	NEDD4-like E3 ubiquitin protein ligase	0.64
SLC2A4	Solute carrier family 2 member 4 (GLUT4)	0.45
SIRT1	NAD-dependent deacetylase sirtuin-1	0.42
CS	Citrate synthase	−0.53
ACO1	Aconitase 1	−0.55
OGDH	Oxoglutarate dehydrogenase	−0.66
PC	Pyruvate carboxylase	−0.69
SDHC	Succinate dehydrogenase complex subunit C	−0.71
PPARGC1B	PPARG coactivator 1 beta	−0.71
GPT	Glutamic-pyruvic transaminase	−0.85
MSTN	Myostatin	−0.98
IGF1	Insulin-like growth factor 1	−1.21
GPT2	Glutamic-pyruvic transaminase 2	−1.26
IGFBP5	Insulin-like growth factor binding protein 5	−1.28
DLGAP1	DLG associated protein 1	−3.55
VTCN1	V-set domain containing T cell activation inhibitor 1	−4.35
PVALB	Parvalbumin	−4.48
SNCB	Synuclein beta	−5.95
TACR1	Tachykinin receptor 1	−6.95

## Data Availability

The raw sequencing data used for the analysis were deposited in the NCBI’s gene expression omnibus public repository with the accession number GSE123929.

## Supplementary Materials

The following supporting information can be downloaded at: https://www.mdpi.com/article/10.3390/ijms242115760/s1.
