# Supplementary material for: Decreased Pyruvate but Not Fatty Acid Driven Mitochondrial Respiration in Skeletal Muscle of Growth Restricted Fetal Sheep
Source: Int J Mol Sci. 2023 Oct 30;24(21):15760. doi: 10.3390/ijms242115760 (PMC10648961; doi:10.3390/ijms242115760)
Supplement: Supplementary file 1 [file ijms-24-15760-s001.zip › Supplementary Table S2.pdf]

Supplementary Table S2. Primer details for the qPCR experiments

Gene name	Forward	Reverse
PDK4	CCC AGA GGA CCA AAA GG CAT	GGG TCA GCT GTA CAG GCA TC
ACADVL	GCA GAG GTG TAC TTT GAC GGA	TCC AAA CTG GGT ACG GTT AGC
CDKN1A	GCC AAC CAG AAC AAC TCA ACC	AAG TGG TCC TCC TGA GAC GTG
HADHB	GGA CTC CTT TCT GAT GTT GTG C	GTT TGG CCA TCT GAT CCA GAG A
IGF1	TCG CAT CTC TTC TAT CTG GCC CTG T	GCA GTA CAT CTC CAG CCT CCT CAG A
PHGDH	AGCAAAGAGGAGCTGATAGCC	TCCCATGAACTTCTTCCGCTC
GADD45G	CGT CTA CGA GTC AGC CAA AGT	CGG GAA GGG TAA TAT TGG GCA
PVALB	TCG GCC TGA AGA AAA AGA GCC	CCA GCA GCA TCT TGG TTT CTT
PSAT1	GGT CAC AGT GGT GAT CGT TCG	CAG GAC CAA GCC CAT GAC ATA
IGFR1	AAC TGT CAT CTC CAA CCT C	CAA GCC TCC CAC TAT CAA C
FOXO3	GAA TTC CGT CAG CAA CAT GGG	GGT TGA GTA CAA GGA GGA GCC
SIRT1	TCG TGC GAA AGT GAC GAA GAT	AAT CGT TCG AGG ATC TGT GCC
IGFBP4	TGT GCG TGT GTG TTG ATG	GAG GGA GCC AAG ATG AGT
IGFBP7	TGG TGC CCA GGT GTA TTT GAG	TCT GAA TGG CCA GGT TGT CTC
GNL2	ACG TTC CCC TCA CAT TGA GAC	AGC AAG TGT CGG GTA ATC CTG
EIF4EBP1	CCG GAG GTA CCA GGA TCA TCT	ATC GCC TGT AGG GCT AGT GA
IGF2BP2	ATA TTT CTC CAG CCT GTG CCC	TCA GTT TCC CAA AGA TCC GCC
IGFBP6	AGG AGA GTA AGC CCC AAG CAG	GTC ACA ATT GGG CAC GTA GAG
IGFBP5	TCG TGC GGC GTC TAC ACT GAG	GAG TAG GTC TCC TCC GCC ATC
Myostatin	GCT GTA ACC TTC CCA GAA CCA	TTT GCT TGG TGC ACA AGA TGG
